# Achieving High Efficiency and Stability in Organic Photovoltaics with a Nanometer-Scale Twin p–i–n Structured Active Layer

**DOI:** 10.1021/acsami.4c08868

**Published:** 2024-07-23

**Authors:** Bin Chang, Bing-Huang Jiang, Chih-Ping Chen, Kai Chen, Bo-Han Chen, Shaun Tan, Tzu-Ching Lu, Cheng-Si Tsao, Yu-Wei Su, Shang-Da Yang, Cheng-Sheng Chen, Kung-Hwa Wei

**Affiliations:** † Department of Materials Science and Engineering, National Yang Ming Chiao Tung University, Hsinchu 30010, Taiwan; ‡ Department of Materials Engineering, Ming Chi University of Technology, New Taipei City 243303, Taiwan; § College of Engineering, Chang Gung University, Taoyuan 33302, Taiwan; ∥ Robinson Research Institute, Victoria University of Wellington, Wellington 6012, New Zealand; ⊥ MacDiarmid Institute for Advanced Materials and Nanotechnology, Wellington 6012, New Zealand; # The Dodd-Walls Centre for Photonic and Quantum TechnologiesUniversity of Otago, Denedin 9016, New Zealand; ∇ Institute of Photonics Technologies, National Tsing Hua University, Hsinchu 300044, Taiwan; ○ Department of Chemistry, Massachusetts Institute of Technology, Cambridge, Massachusetts 02139, United States; ◆ Department of Materials Science and Engineering, National Taiwan University, Taipei 106319, Taiwan; ¶ National Synchrotron Radiation Research Center, Hsinchu 30010, Taiwan; †† Department of Molecular Science and Engineering, Institute of Organic and Polymeric Materials, National Taipei University of Technology, Taipei 10608, Taiwan

**Keywords:** organic photovoltaics, sequential deposition, twin p–i–n structure, thermal stability, morphology

## Abstract

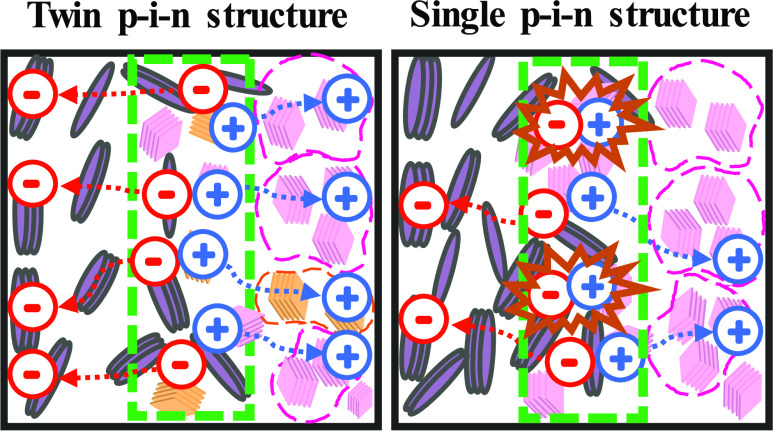

In pursuing high stability and power conversion efficiency for organic photovoltaics (OPVs), a sequential deposition (SD) approach to fabricate active layers with p–i–n structures (where p, i, and n represent the electron donor, mixed donor:acceptor, and electron acceptor regions, respectively, distinctively different from the bulk heterojunction (BHJ) structure) has emerged. Here, we present a novel approach that by incorporating two polymer donors, **PBDBT-DTBT** and **PTQ-2F**, and one small-molecule acceptor, **BTP-3-EH-4Cl**, into the active layer with sequential deposition, we formed a device with nanometer-scale twin p–i–n structured active layer. The twin p–i–n **PBDBT-DTBT:PTQ-2F/BTP-3-EH-4Cl** device involved first depositing a **PBDBT-DTBT:PTQ-2F** blend under layer and then a **BTP-3-EH-4Cl** top layer and exhibited an improved power conversion efficiency (PCE) value of 18.6%, as compared to the 16.4% for the control BHJ **PBDBT-DTBT:PTQ-2F:BTP-3-EH-4Cl** device or 16.6% for the single p–i–n **PBDBT-DTBT/BTP-3-EH-4Cl** device. The PCE enhancement resulted mainly from the twin p–i–n active layer’s multiple nanoscale charge carrier pathways that contributed to an improved fill factor and faster photocurrent generation based on transient absorption studies. The **PBDBT-DTBT:PTQ-2F/BTP-3-EH-4Cl** film possessed a vertical twin p–i–n morphology that was revealed through secondary ion mass spectrometry and synchrotron grazing-incidence small-angle X-ray scattering analyses. The thermal stability (*T*_80_) at 85 °C of the twin p–i–n **PBDBT-DTBT:PTQ-2F/BTP-3-EH-4Cl** device surpassed that of the single p–i–n **PBDBT-DTBT/BTP-3-EH-4Cl** devices (906 vs 196 h). This approach of providing a twin p–i–n structure in the active layer can lead to substantial enhancements in both the PCE and stability of organic photovoltaics, laying a solid foundation for future commercialization of the organic photovoltaics technology.

## 1 Introduction

Organic photovoltaics (OPVs) is a promising renewable energy technology owing to its lightweight, flexibility, low cost, and environmental friendliness in manufacturing.^1−6^ The bulk-heterojunction (BHJ) structure is usually the most prevalent and efficient active layer design in the development of OPVs to date. Owing to a spontaneous phase separation, the BHJ structure typically comprises three phases: randomly oriented and distributed donor (p) domains and acceptor (n) domains that are mutually mixed with each other and molecularly mixed donor-and-acceptor (i) domains, providing extensive interfacial contacts that are within the Frenkel exciton diffusion length for improving charge dissociation. Despite such benefits, the BHJ active layer has a morphology that unintentionally promotes nonradiative recombination processes during the carrier transport process, caused by the randomly distributed p- and n-domains, which leads to limited device efficiency.^7−9^ Moreover, the performance of OPVs depends not only on the degree of mixing between the donor and acceptor but also on the vertical phase separation in the active layer.^10,11^ In a typical BHJ binary blend of a polymer donor and a small-molecule acceptor, the polymer donor tends to aggregate at the surface owing to the relatively low surface energy, as compared with that of the small-molecular acceptor, resulting in an aggregation of the polymer near the cathode, and the small-molecular near the anode in the device structure.^12,13^ This is not an ideal structure because electrons and holes will not always be extracted by the right electrodes. More complicated BHJ ternary blends comprising two donors and one acceptor often have unfavorable interactions between the two donors,^14^ causing severe molecular disorders and large domain sizes that can act as charge recombination trap sites and thereby decreasing the OPV performance.^15^

To date, various processing methods, such as vacuum deposition, film-transfer (floating or using poly(dimethylsiloxane) stamp), or multistep spin-coating processes with orthogonal solvents, have been applied for fabricating OPVs with stacked structures.^16,17^ The sequential deposition (SD),^18−21^ also known as the layer-by-layer process, features donor- and acceptor-layer stacked structures with significant advantages in providing more balanced charge transport after exciton dissociation and direct pathways for carriers, particularly near the electrode regions.^22−26^ As compared to the BHJ structure, the SD method provides independent processing and optimization of donor and acceptor sublayers as well as more controllable morphology that has a pseudo p–i–n structure.^27,28^ The p–i–n active layer typically has a substantially large and well-mixed interfacial (i) middle region, surrounded by two relatively thin but completely layered p- and n-regions (sublayers) without any mixed counter p- or n-domains. The orientation of these p–i–n layers is normal to the carrier transport direction, favorable for charge transport. Better control of the p–i–n active layer morphology in devices is also more ideal for improving the reproducibility during industrial device production. With the development of the SD method, the vertical phase separation can be controlled to promote charge extraction and transport at the appropriate electrodes.^29−32^ By spin-coating the donor and acceptor materials layer-by-layer with post-treatment processes, a p–i–n-like structure can be achieved with aggregated polymer donors near the anode and small-molecular acceptors near the cathode.^33^ On the other, traditional methods for casting ternary blend films result in unfavorable overmixed systems.^34^

This research demonstrates that interdiffusion processes of two polymer donors with one acceptor can be used to create so-called “twin p–i–n structures” through an SD process. For the two donors and one acceptor system, we chose poly[(2,6-(4,8-bis(5-(2-ethylhexyl-3-fluoro)thiophen-2-yl)-benzo[1,2-b:4,5-b’]dithiophene))-*alt*-5,5′-(5,8-bis(4-(2-butyloctyl)thiophen-2-yl)dithieno[3′,2′:3,4;2″,3″:5,6]benzo[1,2-*c*][1,2,5]thiadiazole)] (**PBDBT-DTBT**) and poly [[6,7-difluoro[(2-hexyldecyl)oxy]-5,8-quinoxalinediyl]-2,5-thiophenediyl ] (**PTQ-2F**) as the two donors, and 2,2′-((2Z,2′Z)-((12,13-bis(3-ethylheptyl)-3,9-diundecyl-12,13-dihydro-[1,2,5]thiadiazolo[3,4-*e*]thieno[2″,3″:4′,5′]thieno[2′,3′:4,5]pyrrolo[3,2-*g*]thieno[2′,3′:4,5]thieno[3,2-*b*]indole-2,10-diyl)bis(methanylylidene))bis(5,6-dichloro-3-oxo-2,3-dihydro-1H-indene-2,1-diylidene))dimalononitrile (**BTP-3-EH-4Cl**) as the acceptor for processing OPVs with either the SD or BHJ active layers. We fabricated the twin p–i–n structure active layer by first spin-casting a solution composed of mixed **PBDBT-DTBT:PTQ-2F** donors followed by a solution of **BTP-3-EH-4Cl** acceptor. We carried out morphological characterizations that clearly demonstrate the nanoscale landscape of the twin p–i–n structure, as determined by the preaggregation kinetics of the upper layer (acceptor) and interdiffusion. We also performed carrier dynamics and energy loss analyses that showed improved hole transfer (HT) dynamics and inhibited trap states, both contributing to the simultaneous enhancement of device *V*_OC_. With these advantages, highly efficient devices with the twin p–i–n **PBDBT-DTBT:PTQ-2F/BTP-3-EH-4Cl** active layer achieved a power conversion efficiency (PCE) of 18.6%, higher than the 16.4 and 16.6% for the devices with a BHJ **PBDBT-DTBT:PTQ-2F:BTP-3-EH-4Cl** ternary blend active layer, or a single p–i–n **PBDBT-DTBT/BTP-3-EH-4Cl** active layer, respectively. Simultaneously achieving both high PCE and good stability for emerging OPVs has been a significant challenge, and in this study, we also performed thermal aging of the samples at 85 °C. Significantly, the *T*_80_ lifetime of the **PBDBT-DTBT:PTQ-2F/BTP-3-EH-4Cl** device is 906 h, far surpassing the 196 h (**PBDBT-DTBT/BTP-3-EH-4Cl** device) and 284 h (**PTQ-2F/BTP-3-EH-4Cl** device) of the control devices. The improvement is attributed to the large domain size in the twin p–i–n structured **PBDBT-DTBT:PTQ-2F/BTP-3-EH-4Cl** device that provides a favorable network for slowing the morphological degradation during the aging process.

## 2 Experimental Section

### 2.1 Fabrication of Organic Photovoltaics (OPV) Devices

Two polymers **PBDBT-DTBT** and **PTQ-2F** and one small-molecule **BTP-3-EH-4Cl** were purchased from Derthon OPV (purity >99%). Zinc oxide nanoparticle (ZnO NP) inks were purchased from Sigma (2.5 wt %, viscosity 2.1 cP, work function −3.9 eV), and poly(3,4-ethylenedioxythiophene):polystyrenesulfonate (PEDOT:PSS; Clevios P VP Al 4083) was purchased from Heraeus. OPV devices were fabricated with the conventional structure of glass/indium tin oxide (ITO)/PEDOT:PSS/Active layer [single or twin p-i-n structure]/ZnO NP/Ag. The fabrication steps involve that the ITO-coated glass (approximately 5 Ω^–1^) was first cleaned using a detergent, deionized water, acetone (purity > 99%), and isopropyl alcohol (purity >99%) where each case was subjected to ultrasonication (20 min), followed by drying in an oven (60 min). Then, the cleaned substrates were treated with ultraviolet-ozone (UVO) for 15 min. After spin-coating PEDOT:PSS onto the ITO (at 4500 rpm for 30 s) and annealing the substrates in air (at 150 °C for 20 min), they were placed in an N_2_-filled glovebox. To prepare OPV devices with the single p–i–n architecture, a solution containing either the donor PBDBT-DTBT or PTQ-2F in chloroform (CF, purity > 99%; 5 or 10 mg mL^–1^) was spin-coated onto the PEDOT:PSS at 3000 rpm for 40 s. For the twin p–i–n architecture, a solution of the PBDBT-DTBT:PTQ-2F donor blend (at a ratio of 9:1 in CF; 5 mg mL^–1^) was spin-coated onto the PEDOT:PSS at 2750 rpm for 40 s, forming the front layer. Finally, the acceptor BTP-3-EH-4Cl in CF (10 mg mL^–1^) was spin-coated onto the deposited and dried donor layer at 3000 rpm for 40 s. These solutions were individually prepared by stirring for 3 h at 60 °C in a glovebox and then cooling to room temperature (around 25 °C) before the active layer process. ZnO NPs were spin-coated (4000 rpm, 60 s) onto the active layer as the ETL. The SD fabrication of the OPVs was completed by thermally evaporating a 100 nm-thick Ag film under high vacuum (approximately 10^–7^ Torr; with a shadow mask area of 0.1 cm^2^). The Ag slug purity is >99.995%.

### 2.2 Characterizations

Absorption and transmittance spectra were recorded by using a UV–Vis spectrophotometer (Hitachi U-4100). A Keithley 2400 source meter was used to record the current density–voltage (*J*–*V*) characteristics. Photocurrents were recorded using a Xe lamp–based 150-W solar simulator (simulated AM 1.5G illumination; 100 mW cm^–2^). The illumination intensity was confirmed using a calibrated Si photodiode and a KG-5 filter. An integrated system (Enlitech, Taiwan) was used to determine the external quantum efficiencies (EQEs), with a calibrated monosilicon diode (responding in the range of 300–1000 nm) as a reference. The photocurrent (*J*_ph_/*J*_sat_) with respect to the effective voltage (*V*_eff_ = *V*_0_–*V*), where *J*_sat_ represents the saturation current density, *V*_0_ is the voltage when photocurrent density (*J*_ph_) value equals 0, and *V* is the applied voltage. Film morphologies were measured under ambient conditions using tapping-mode atomic force microscopy (AFM; Veeco Innova). Transient absorption (TA) measurements were conducted by using a homemade system that can flexibly support excitation at 850 nm and probe spectrum across 550–1000 nm. The mobilities were extracted by fitting the Space Charge-Limited Current (SCLC) model to the *J*–*V* curves measured in the dark. The hole-only diodes were fabricated as the ITO/PEDOT:PSS/active layer/Au, and electron-only diodes were fabricated as the ITO/ZnO/active layer/Al. Grazing-incidence wide-angle X-ray scattering (GIWAXS) (incident angle: 0.5°) and grazing-incidence small-angle X-ray scattering (GISAXS) (incident angle: 0.2°) were performed using the 23A beamline (10 keV) at the National Synchrotron Radiation Research Center (NSRRC), Hsinchu, Taiwan. We conducted GIWAXS and GISAXS measurements by the active layer spin-coating on a Si wafer to accurately observe the morphology of the active layer. A flat-panel detector (pixel number of C10158DK is 2352) was used to collect the wide-angle scattering signals with a sample-to-detector distance of 19.5 cm, and the CCD MAR 165 was used to collect the small-angle scattering signals with a sample-to-detector distance of 281 cm. A thermal aging test was performed on a temperature-controlled hot plate at 85 °C in a dark glovebox under an inert atmosphere (O_2_ < 0.1 ppm, H_2_O < 0.1 ppm).

## 3 Results and Discussion

### 3.1 Characterization of Optoelectrical Properties

**Figure 1:**
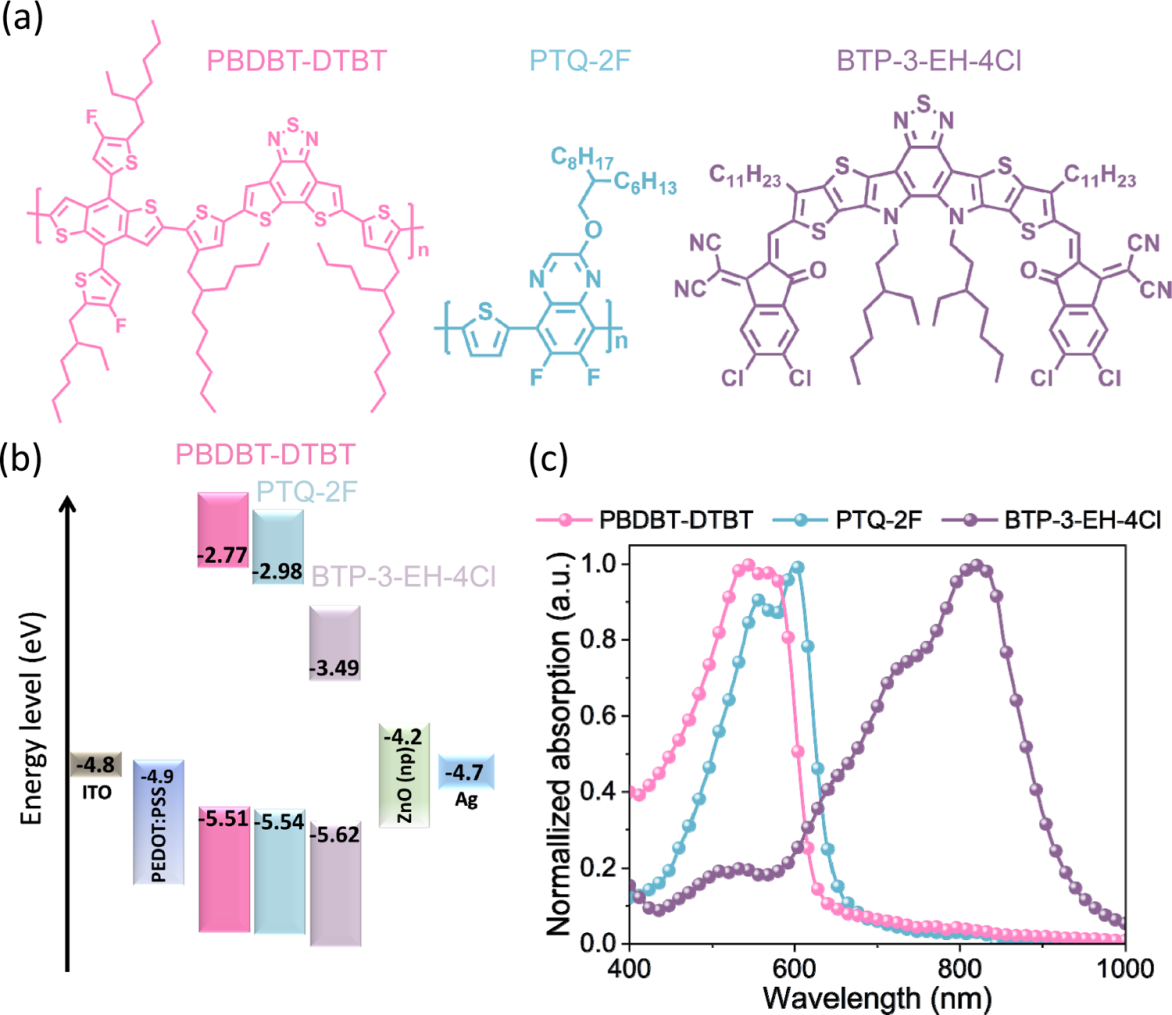
Basic properties of the various materials used in the active layer used in this study. (a) Chemical structure, (b) energy levels, and (c) UV–vis spectra of the solid films.

Figure 1a displays the chemical structure of two p-type wide bandgap polymer donors **PBDBT-DTBT** and **PTQ-2F** (two donors) and one narrow bandgap acceptor **BTP-3-EH-4Cl**, which were selected as the active layer for fabricating OPVs. Figure S1 reveals an SD process for first spin-casting a p-type layer composed of a blend of the two polymer donors **PBDBT-DTBT** and **PTQ-2F**, followed by the n-type small-molecule acceptor **BTP-3-EH-4Cl** onto the p-type layer. The thin-film active layer exhibits a twin p–i–n structure (i referring to an intermixed donor and acceptor layer), created from spontaneous mutual molecular diffusion of the p- and n-type component sublayers during the solution phase. Figure 1b shows the energy levels of the SD-processed devices, which are consistent with previous reports in the literature.^35−37^**PTQ-2F** serving as a guest donor has a shallower LUMO level than **BTP-3-EH-4Cl**, being beneficial for raising the open-circuit voltage (*V*_OC_) of the device. Figure 1c presents the UV–vis absorption spectra of **PBDBT-DTBT, PTQ-2F**, and **BTP-3-EH-4Cl** neat films; it can be seen that **BTP-3-EH-4Cl** has a maximum absorption wavelength at 800 nm, complementary to those of **PBDBT-DTBT** and **PTQ-2F**. In Figure S2, the twin p–i–n structured **PBDBT-DTBT:PTQ-2F/BTP-3-EH-4Cl** film shows an enhanced absorption coefficient, which is helpful to increase the light harvesting and thereby the photocurrent of the device.

**Figure 2:**
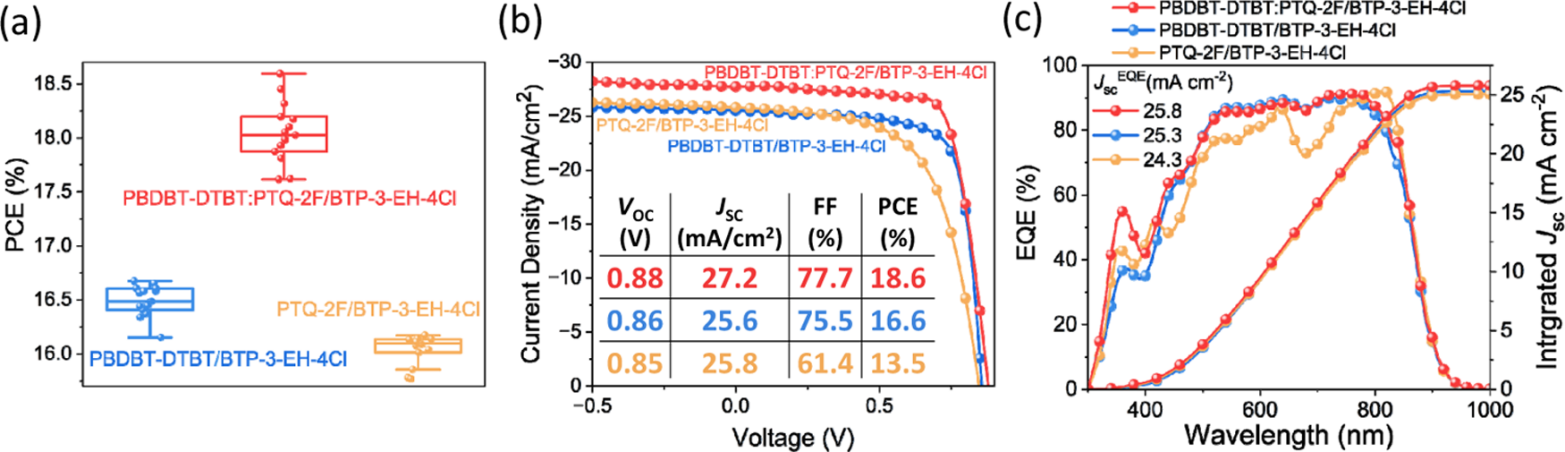
(a) Box plots showing the distribution of the PCEs of the devices. (b) Current density–voltage curves of the best-performing devices. The inset includes the measured photovoltaic properties. (c) EQE spectrum and integrated *J*_SC_ of the devices.

Figure 2a shows the box plot of the PCE distribution for various devices; Figure 2b depicts current density–voltage (*J*–*V*) curves of the champion devices, with their respective photovoltaic parameters in Table 1. The device with the SD-processed **PBDBT-DTBT/BTP-3-EH-4Cl** active layer had a *V*_OC_ value of 0.86 V, a *J*_SC_ value of 25.6 mA cm^–2^, and a fill factor (FF) value of 75.5%, resulting in a PCE of 16.6%. On the other hand, the device with SD-processed **PTQ-2F/BTP-3-EH-4Cl** active layer had *V*_OC_, *J*_SC_, and FF values of 0.85 V, 25.8 mA cm^–2^, and 61.4%, respectively, with a PCE value of 13.5%. On the other hand, the device comprising the twin p–i–n structured **PBDBT-DTBT:PTQ-2F/BTP-3-EH-4Cl** active layer displayed the best PCE of 18.6%, with *V*_OC_, *J*_SC_, and FF values of 0.88 V, 27.2 mA cm^–2^, and 77.7%, respectively. The PCE values of the devices follow the trend of **PBDBT-DTBT:PTQ-2F/BTP-3-EH-4Cl** > **PBDBT-DTBT/BTP-3-EH-4Cl** > **PTQ-2F/BTP-3-EH-4Cl**, owing to the different values of *V*_OC_, *J*_SC_, and FF. On the other hand, OPVs with the regular BHJ structure and blends of **PBDBT-DTBT:PTQ-2F:BTP-3-EH-4Cl, PBDBT-DTBT:BTP-3-EH-4Cl**, and **PTQ-2F:BTP-3-EH-4Cl** exhibit PCE values of 16.4, 16.1, and 7.6%, respectively, all lower than that of the corresponding devices with the p–i–n active layer, especially for the devices with an active layer involving the BHJ blend of **PTQ-2F** and **BTP-3-EH-4Cl**. Figure S5 shows the PCE distribution of the **PBDBT-DTBT:PTQ-2F/BTP-3-EH-4Cl** devices with different ratios of **PBDBT-DTBT:PTQ-2F**.

**Table 1:** Photovoltaic Device Parameters of SD and BHJ Devices^a^

active layer configuration	structure	*V*_oc_ (V)	*J*_sc_ (mA/cm^2^)	FF (%)	PCE (%)
**PBDBT-DTBT:PTQ-2F/BTP-3-EH-4Cl**^b^	SD^c^	0.88 ± 0.001	26.5 ± 0.40	77.5 ± 0.28	18.0 ± 0.3
**PBDBT-DTBT:PTQ-2F:BTP-3-EH-4Cl**^b^	BHJ	0.86 ± 0.01	27.1 ± 0.49	70.8 ± 0.19	16.4 ± 0.5
**PBDBT-DTBT/BTP-3-EH-4Cl**	SD^c^	0.86 ± 0.010	25.6 ± 0.22	75.5 ± 0.28	16.5 ± 0.1
**PBDBT-DTBT:BTP-3-EH-4Cl**	BHJ	0.86 ± 0.01	26.6 ± 0.72	70.7 ± 0.02	16.1 ± 0.7
**PTQ-2F/BTP-3-EH-4Cl**	SD^c^	0.85 ± 0.001	25.8 ± 0.24	61.4 ± 0.26	13.2 ± 0.1
**PTQ-2F:BTP-3-EH-4Cl**	BHJ	0.87 ± 0.01	15.8 ± 1.19	55.8 ± 0.07	7.6 ± 1.1

a Fifteen Devices were Fabricated in Each Case.

b Weight ratio of **PBDBT-DTBT**:**PTQ-2F** = 95:5.

c The thickness of D/A layers is 40/60 nm.

Figure 2c shows the calculated *J*_SC_ values (*J*_SC_^EQE^) based on integrating the EQE spectra of the **PBDBT-DTBT:PTQ-2F/BTP-3-EH-4Cl, PBDBT-DTBT/BTP-3-EH-4Cl**, and **PTQ-2F/BTP-3-EH-4Cl** devices, giving 25.9, 25.3, and 24.3 mA cm^–2^, respectively, matching relatively closely (<5% deviation) with the *J*_SC_ values obtained from the corresponding *J*–*V* curves with 27.2, 25.6, and 25.8 mA cm^–2^. The EQE curves show that the **PBDBT-DTBT:PTQ-2F/BTP-3-EH-4Cl** device exhibited the highest EQE value in the range from 400 to 850 nm, as compared to that of the **PTQ-2F/BTP-3-EH-4Cl** and **PBDBT-DTBT/BTP-3-EH-4Cl** devices, and this enhancement can be attributed to the significantly larger optical absorption coefficients of the **PBDBT-DTBT:PTQ-2F/BTP-3-EH-4Cl** active layer than those of the **PTQ-2F/BTP-3-EH-4Cl** and **PBDBT-DTBT/BTP-3-EH-4Cl** active layers. Table 2 lists the champion photovoltaic device parameters of SD devices and the corresponding *J*_sc_ values integrated from the EQE curves.

**Table 2:** Champion Photovoltaic Device Parameters of SD Devices and the Corresponding *J*_sc_ Values Integrated from the EQE Curves

active layer configuration	*V*_oc_ (V)	*J*_sc_ (mA/cm^2^)	*J*_sc_^EQE^	FF (%)	PCE (%)
**PBDBT-DTBT:PTQ-2F/BTP-3-EH-4Cl**	0.88	27.2	25.9	77.7	18.6
**PBDBT-DTBT/BTP-3-EH-4Cl**	0.86	25.6	25.3	75.7	16.6
**PTQ-2F/BTP-3-EH-4Cl**	0.85	25.8	24.3	61.4	13.5

The FF values of these **PTQ-2F/BTP-3-EH-4Cl**, **PBDBT-DTBT/N3–4C**, and **PBDBT-DTBT:PTQ-2F/BTP-3-EH-4Cl** devices varied substantially from 61.4 to 77.7%, with the largest FF value appearing in the case of the **PBDBT-DTBT:PTQ-2F/BTP-3-EH-4Cl** device, suggesting that the twin p–i–n active layer has an advantageous morphology via aggregation features.^38^ Since the FF change was the major contributing factor to the PCE variations in the devices, we attempted to rationalize the FF trends by studying the charge transport processes through the technique of photoinduced charge carrier extraction with a linearly increasing voltage, referred to as the photo-CELIV method.^39,40^ The carrier mobility (μ) of the **PTQ-2F/BTP-3-EH-4Cl, PBDBT-DTBT/BTP-3-EH-4Cl**, and **PBDBT-DTBT:PTQ-2F/BTP-3-EH-4Cl** devices was analyzed by using the photo-CELIV method (shown in Figure S3). The extracted μ value for the twin p–i–n **PBDBT-DTBT:PTQ-2F/BTP-3-EH-4Cl** device is 1.66 × 10^–4^ cm^2^ V^–1^s^–1^, superior to those of the single p–i–n **PBDBT-DTBT/BTP-3-EH-4Cl** and **PTQ-2F/BTP-3-EH-4Cl** devices that are 1.55 × 10^–4^ and 1.34 × 10^–4^ cm^2^ V^–1^s^–1^, respectively. Moreover, we carried out further investigations to confirm the trend in the charge mobility of the devices by employing SCLC measurements (Figure S4). Our results reveal that the hole-to-electron mobility ratio of the twin p–i–n **PBDBT-DTBT:PTQ-2F/BTP-3-EH-4Cl** device is 1.02, while the ratio for the single p–i–n **PBDBT-DTBT/BTP-3-EH-4Cl** and **PTQ-2F/BTP-3-EH-4Cl** devices are 1.24 and 0.8, respectively, indicating that the twin p–i–n structure has more balanced charge transport.^41,42^ In summary, the OPV with the twin p–i–n structured active layer involving a vertical distribution of donor and acceptor molecules led to enhanced charge transfer and more balanced charge mobility, resulting in the highest PCE of 18.6% among all tested devices.

### 3.2 Carrier Lifetime and Energy Loss in Devices

**Figure 3:**
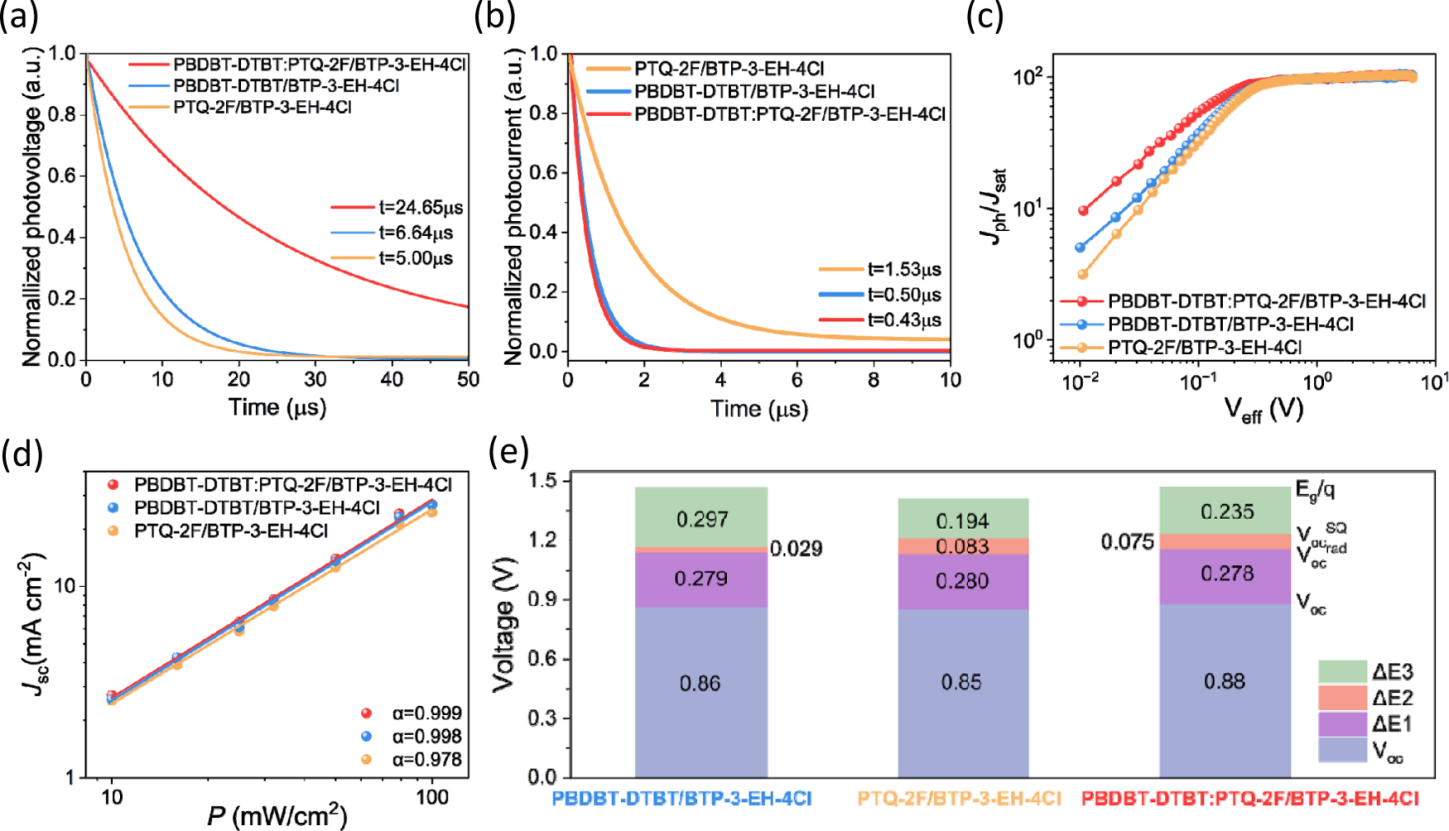
Facilitated charge extraction, exciton dissociation, carrier recombination process, and energy loss. (a) Transient photovoltage. (b) Transient photocurrent curves (au, arbitrary units). (c) Photocurrent data as a function of effective voltage *V*_eff_. (d) Dependence of *J*_SC_ on light intensity. (e) Detailed energy loss of the OPV devices.

Light-intensity transient photovoltage measurements were conducted (Figure 3a) to probe the charge recombination dynamics in our devices. The charge carrier lifetime (τ) for the twin p–i–n structured **PBDBT-DTBT:PTQ-2F/BTP-3-EH-4Cl** device is 24.65 μs, being significantly longer than the 6.64 μs for the **PBDBT-DTBT/BTP-3-EH-4Cl** device and 5.00 μs for the **PTQ-2F/BTP-3-EH-4Cl** device, indicating a reduced carrier recombination rate in the twin p–i–n structured device compared to that of the single p–i–n devices. We also measured the transient photocurrent curves (Figure 3b) of the p–i–n structured OPVs to obtain the charge carrier extraction of the corresponding devices. The twin p–i–n **PBDBT-DTBT:PTQ-2F/BTP-3-EH-4Cl** device displayed the shortest decay time of 0.43 μs, indicating fast charge extraction from the active layer to the electron transport layer.^43,44^ The accelerated charge extraction suggests a reduced carrier recombination at the interface, which can also account for the higher *J*_sc_ observed in the twin p–i–n **PBDBT-DTBT:PTQ-2F/BTP-3-EH-4Cl** devices from the *J*–*V* tests.^45^ From the carrier mobility and lifetime analyses results, we suspect that the superior performances of the twin p–i–n **PBDBT-DTBT:PTQ-2F/BTP-3-EH-4Cl** device are attributed to the twin p–i–n structured active layer that provides improved charge extraction, carrier transport, and reduced nonradiative recombination. In addition, we believe that the more suitable crystallinity and surface morphology of the twin p–i–n **PBDBT-DTBT:PTQ-2F/BTP-3-EH-4Cl** active layer also contributed to the improved device performance.^46^Figure 3c shows a plot of the corrected photocurrent (*J*_ph_/*J*_sat_) with respect to the effective voltage (*V*_eff_ = *V*_0_–*V*), where *J*_sat_ represents the saturation current density, *V*_0_ is the voltage when photocurrent density (*J*_ph_) value equals 0, and *V* is the applied voltage. At low values of *V*_*e*ff_ (ca. 0.01–0.5 V), the twin p–i–n **PBDBT-DTBT:PTQ-2F/BTP-3-EH-4Cl** device had the highest *J*_ph_/*J*_sat_ value among all cases.^47^ Both results suggest that the twin p–i–n **PBDBT-DTBT:PTQ-2F/BTP-3-EH-4Cl** device had charge separation superior to that of the **PBDBT-DTBT/BTP-3-EH-4Cl** and **PTQ-2F/BTP-3-EH-4Cl** devices, likely owing to the favorable vertical distribution of its twin p–i–n architecture. Figure 3d illustrates the relationship between *J*_SC_ and incident light power (*P*), where *J*_SC_ is proportional to *P*^α^. As α approaches 1, charge carriers in the active layer in the devices display the lowest degree of bimolecular recombination. Compared to the device with single p–i–n structures (**PBDBT-DTBT/BTP-3-EH-4Cl** and **PTQ-2F/BTP-3-EH-4Cl**), the device with the twin p–i–n structured **PBDBT-DTBT:PTQ-2F/BTP-3-EH-4Cl** provided the highest α value of 0.999, suggesting dramatically suppressed bimolecular recombination. Also, Figure S6 illustrates the relationship between *V*_OC_ and *P*,^48^ along with the *V*_OC_ value proportional to (*nkT*/*q*) ln(*P*), where *q*, *k*, and *T* represent the elementary charge, Boltzmann’s constant, and absolute temperature, respectively. The higher *n* value indicates greater trap-assisted charge recombination in the active layer. The *n* values of **PBDBT-DTBT/BTP-3-EH-4Cl**, **PTQ-2F/BTP-3-EH-4Cl**, and **PBDBT-DTBT:PTQ-2F/BTP-3-EH-4Cl** are 1.16, 1.31, and 1.09, respectively, indicating that the twin p–i–n structured **PBDBT-DTBT:PTQ-2F/BTP-3-EH-4Cl** device has higher degree of charge extraction, with suppressed trapped-assisted charge recombination. We also probed the energy loss (*E*_loss_) of the devices using Fourier-transform photocurrent spectroscopy-external quantum efficiency (FTPS-EQE) (Figure S7a–c) and electroluminescence-EQE (EL-EQE) experiments (Figure S7d).^47−48,49,50^ The total energy loss (*E*_loss_) for a device can be defined as a combination of the energy loss from radiative recombination loss (Δ*E*_1_), the energy loss from charge generation (Δ*E*_2_ = *E*_gap_ – *E*_CT_), and the energy loss from nonradiative recombination loss [Δ*E*_3_ = −*kT* ln(EQE_EL_)]; the *E*_loss_ can be calculated based on Equation
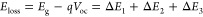
1where *k* is the Boltzmann constant, *T* is the absolute temperature, and *q* is the elementary charge. Figure S7a–c shows the fitting curves and experimental data of the FTPS-EQE and EQE_EL_ curves for the devices with either the single p–i–n structured **PBDBT-DTBT/BTP-3-EH-4Cl** and **PTQ-2F/BTP-3-EH-4Cl** or twin p–i–n structured **PBDBT-DTBT:PTQ-2F/BTP-3-EH-4Cl** active layers. The energy loss parameters (*V*_oc_, Δ*E*_1_, Δ*E*_2_, and Δ*E*_3_) are presented in Figure 3e and Table S1. Additionally, the **PTQ-2F/BTP-3-EH-4Cl** device demonstrates lower measured Δ*E*_3_, indicating reduced losses from nonradiative recombination.^51^ Various factors such as recombination through traps, structural defects, triplet states, and Auger recombination contribute to Δ*E*_3_.^52^ Given the identical processing steps of our devices that were manufactured through SD, the higher nonradiative energy loss is likely attributable to traps or structural defects, consistent with findings from our charge carrier recombination studies.^53^

### 3.3 Photocurrent Generation Dynamics

**Figure 4:**
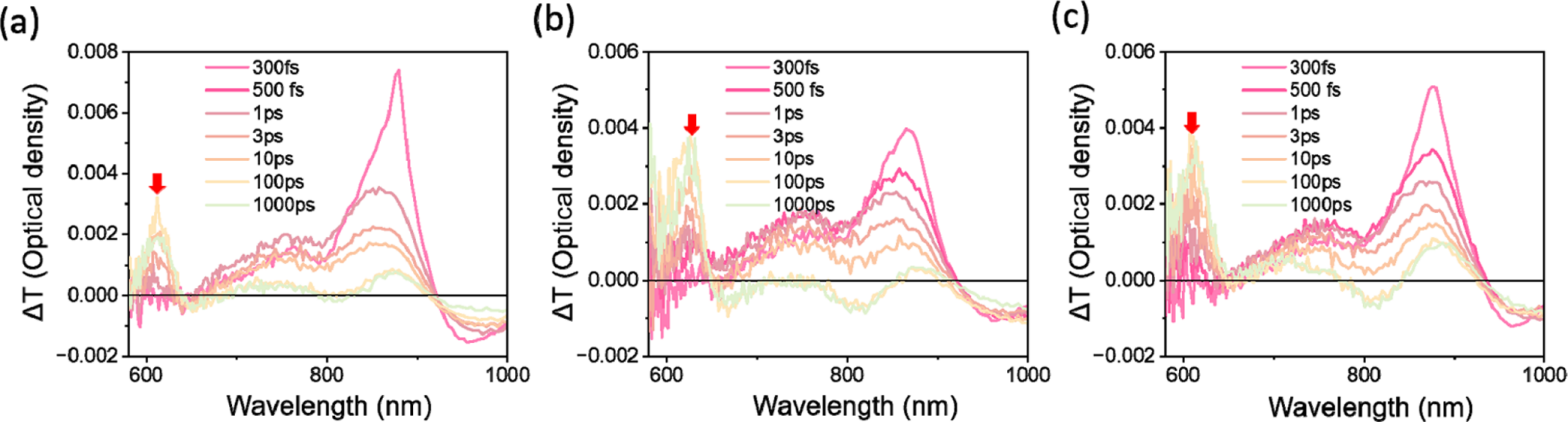
TA spectra of (a) **PBDBT-DTBT/BTP-3-EH-4Cl**, (b)**PTQ-2F/BTP-3-EH-4Cl**, and (c) **PBDBT-DTBT:PTQ-2F/BTP-3-EH-4Cl** at different delay times.

We used Femtosecond transient absorption (TA) spectra to investigate the exciton-charge transfer processes in the films. The hole transfer signal from **BTP-3-EH-4Cl** to the donor can be observed when exciting the films with 850 nm wavelength light, based on the absorption regions of the **PBDBT-DTBT, PTQ-2F**, and **BTP-3-EH-4Cl** molecules. Figure 4a–**c** show that the TA spectra of the **PBDBT-DTBT/BTP-3-EH-4Cl**, **PTQ-2F/BTP-3-EH-4Cl**, and twin p–i–n structured **PBDBT-DTBT:PTQ-2F/BTP-3-EH-4Cl** films exhibit strong ground-state bleaching (GSB) signals of the acceptor in the 620 to 900 nm range. Simultaneously, within subto-picosecond time scales, there is a gradual increase in the GSB signals within the donor’s absorption range in the 550 to 670 nm range (indicated by the red arrow). Moreover, based on the TA spectra at different delay times in Figure 4a–c, the decay of the 850 nm GSB signal of **BTP-3-EH-4Cl** correlates well with the increase of the 640 nm GSB signal of PBDBT-DTBT in the blend films. These signals stem from the effective hole transfer process from **BTP-3-EH-4Cl** to the donor’s domains. Figure S8 shows the corresponding 2D patterns. Comparing with the single p–i–n structured **PBDBT-DTBT/BTP-3-EH-4Cl**, the similar TA dynamics in the twin p–i–n structured **PBDBT-DTBT:PTQ-2F/BTP-3-EH-4Cl** film suggests that the guest donor (**PTQ-2F**) does not introduce recombination centers in the p-type and BHJ regions within nanosecond time scales.^54,55^ Furthermore, the exciton dynamics of our SD devices exhibit characteristics similar to those observed in BHJ devices.^56−58^ This implies that the device with the twin p–i–n structured **PBDBT-DTBT:PTQ-2F/BTP-3-EH-4Cl** active layer not only alleviates charge recombination but also promotes rapid photocurrent generation.

### 3.4 Distribution of the Active Layer Components

**Figure 5:**
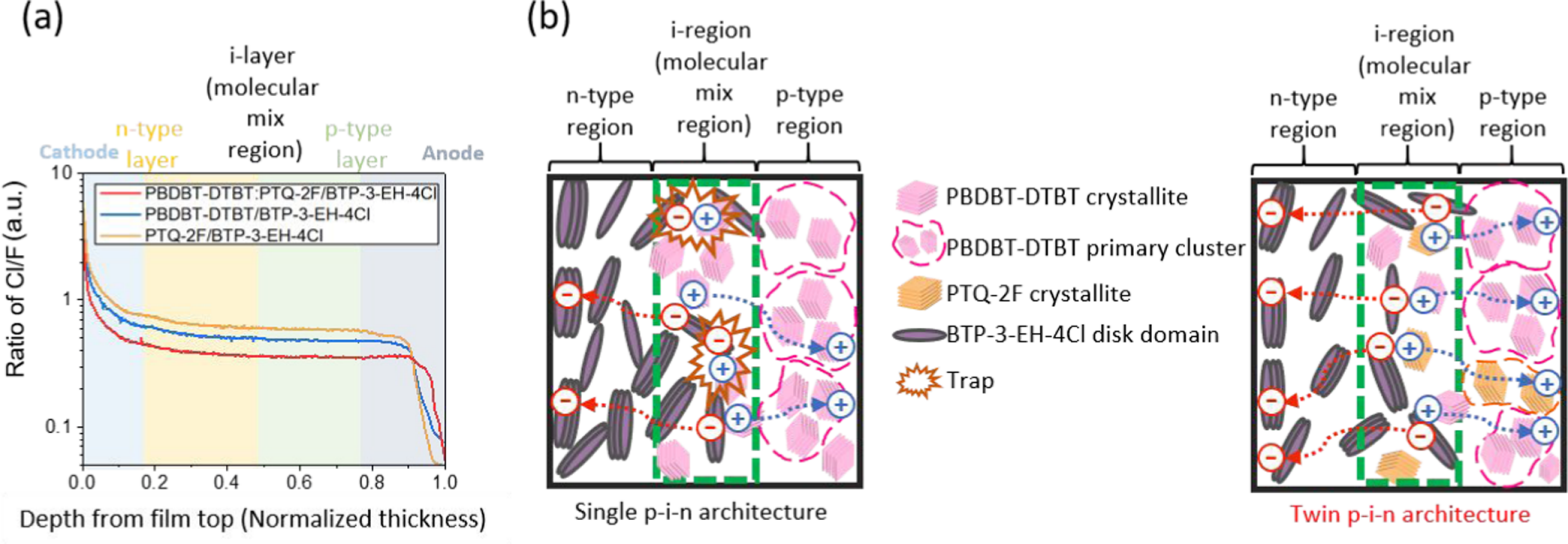
(a) TOF-SIMS yield of Cl/F ratios over sputtering time for **PBDBT-DTBT/BTP-3-EH-4Cl**, **PTQ-2F/BTP-3-EH-4Cl**, and **PBDBT-DTBT:PTQ-2F/BTP-3-EH-4Cl** active layers. (b) Schematic diagram of the single p–i–n structure **PBDBT-DTBT/BTP-3-EH-4Cl** and twin p–i–n structure **PBDBT-DTBT:PTQ-2F/BTP-3-EH-4Cl** active layers.

Figure 5a shows time-of-flight secondary ion mass spectrometry (TOF–SIMS) depth profiles for the single p–i–n **PTQ-2F/BTP-3-EH-4Cl** and **PBDBT-DTBT/BTP-3-EH-4Cl** and twin p–i–n **PBDBT-DTBT:PTQ-2F/BTP-3-EH-4Cl** films, where the total films thicknesses (t) are normalized to be one (t is between 0.0, film top, and 1.0, film bottom). Since only **BTP-3-EH-4Cl** has Cl atoms and both **PBDBT-DTBT** and **PTQ-2F** have only F atoms, the vertical distribution of the **BTP-3-EH-4Cl** acceptor in the films can be probed through the vertical concentration profile by calculating the intensity ratio of Cl to F. In comparing the TOF–SIMS profiles from the top (*t* = 0.0, close to the cathode) to the bottom (*t* = 1.0, close to the anode in the device) of the three films, it is observed that the three films show a common trend that from the top of the films (*t* = 0), the Cl/F signal intensity sharply decreases in the region between *t* = 0.0 and 0.13, which is near the cathodes, and is defined as the n-region (**BTP-3-EH-4Cl** contains only Cl atoms and **PBDBT-DTBT** and **PTQ-2F** both contain F atoms). Moreover, the Cl/F signal intensity plateaus between *t* = 0.13 and 0.88 (about 75% relative thickness), indicating a molecularly intermixed i-region, followed by a sharply decreasing Cl/F signal intensity in the region between *t* = 0.88 and 1.0 near the anode that demarcates the p-region.^59^ In other words, a wide i-region spanning about 75% of the total thickness of the film is surrounded by two relatively thin n- and p-regions (13 and 12% relative thicknesses, respectively). The twin p–i–n structured **PBDBT-DTBT:PTQ-2F/BTP-3-EH-4Cl** films exhibit the lowest ratio value of Cl/F, lower than those of both the single p-i-n structured **PBDBT-DTBT/BTP-3-EH-4Cl** and **PTQ-2F/BTP-3-EH-4Cl**, owing to the higher F concentration in the twin p–i–n **PBDBT-DTBT:PTQ-2F/BTP-3-EH-4Cl** active layer. Additionally, the Cl/F signal intensity ratio in the twin p–i–n structured **PBDBT-DTBT:PTQ-2F/BTP-3-EH-4Cl** film is larger compared to the single structured films at the bottom near *t* = 1.0, indicating a higher relative concentration of **BTP-3-EH-4Cl** in the twin p–i–n structured **PBDBT-DTBT:PTQ-2F/BTP-3-EH-4Cl** films than that in the single p–i–n **PBDBT-DTBT/BTP-3-EH-4Cl** structured films, which is advantageous for exciton dissociation and charge transport. Figure 5b illustrates a schematic drawing of the **PBDBT-DTBT/BTP-3-EH-4Cl** single p–i–n structure (left side) and the **PBDBT-DTBT:PTQ-2F/BTP-3-EH-4Cl** twin p–i–n structure (right side). Using **PTQ-2F** as the second donor in the twin p–i–n structure can contribute additional pathways (a second p-i-n structure) for charge transport that minimizes charge recombination, which contributes to the enhanced OPV performance.

### 3.5 Stability of the Twin p–i–n Structure

**Figure 6:**
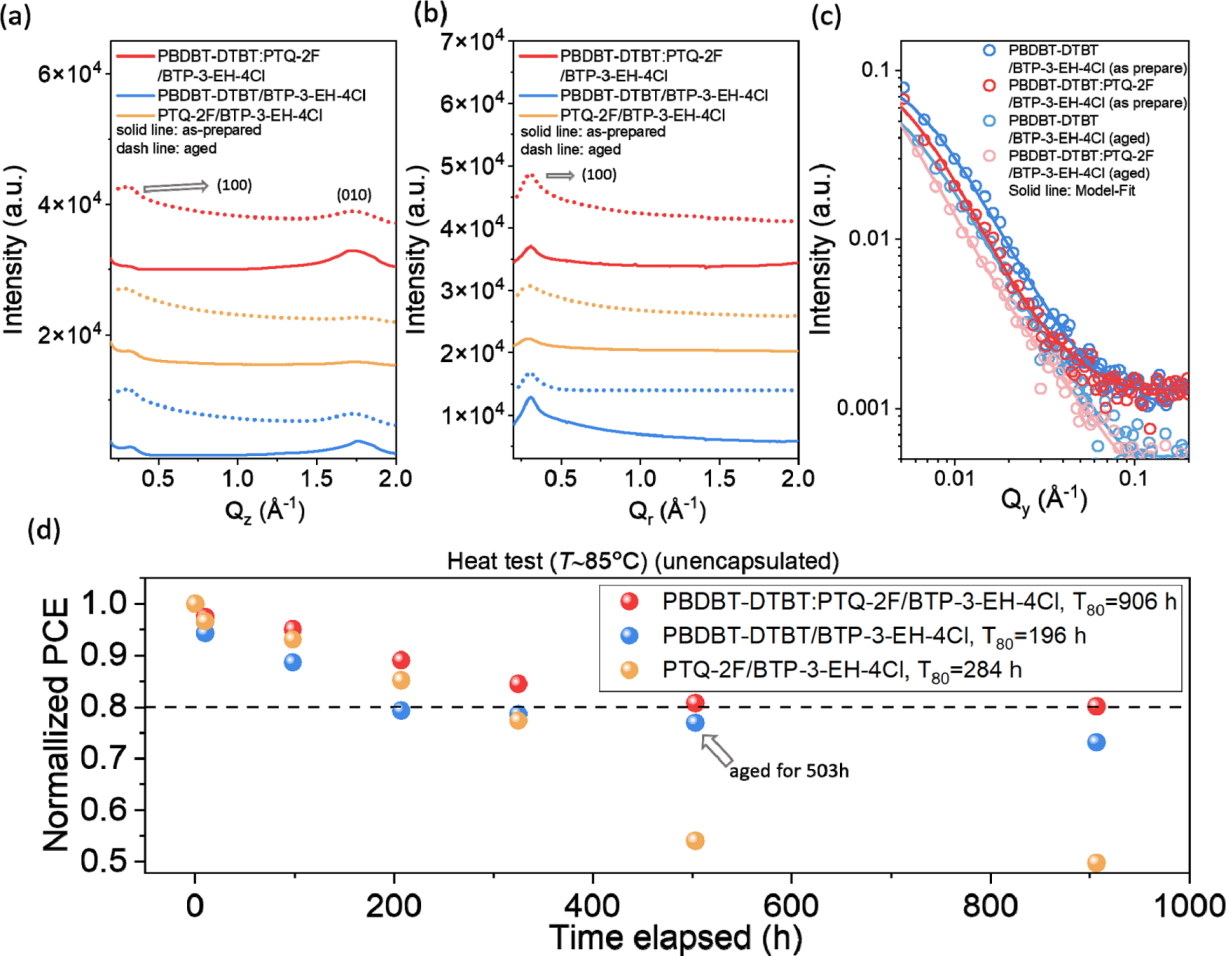
1D GIWAXS profiles in (a) out-of-plane and (b) in-plane of **PBDBT-DTBT/BTP-3-EH-4Cl**, **PTQ-2F/BTP-3-EH-4Cl**, and **PBDBT-DTBT:PTQ-2F/BTP-3-EH-4Cl** as-deposited and aged films (heating under 85 °C for 503 h), along with the corresponding (c) GISAXS profiles. 1D GISAXS profiles and model fitting of the **PBDBT-DTBT/BTP-3-EH-4Cl**, **PTQ-2F/BTP-3-EH-4Cl**, **PBDBT-DTBT:PTQ-2F/BTP-3-EH-4Cl**. (d) Thermal stability of the devices with **PBDBT-DTBT/BTP-3-EH-4Cl**, **PTQ-2F/BTP-3-EH-4Cl**, and **PBDBT-DTBT:PTQ-2F/BTP-3-EH-4Cl** active layers under 85 °C.

The morphology of the active layer is critical to the device performance and stability and can be tuned by various post-treatments, providing insights into the hierarchical structure at multilength scales from the molecular to nanoscale. We investigated the change of the active layer morphology that implies the stability of the devices using simultaneous grazing-incidence wide- and small-angle X-ray scattering (GIWAXS/GISAXS) on the same sample spots^60−68^ at a fixed time. For example, we probed the morphology of the **PBDBT-DTBT/BTP-3-EH-4Cl**, **PTQ-2F/BTP-3-EH-4Cl**, and **PBDBT-DTBT:PTQ-2F/BTP-3-EH-4Cl** p–i–n structured films during their fresh as-prepared states and their corresponding aged degraded states (heating under 85 °C for 503 h). Figure 6a,b presents the one-dimensional (1D) GIWAXS profiles extracted from the two-dimensional (2D) GIWAXS patterns (see Figure S9) of the **PBDBT-DTBT/BTP-3-EH-4Cl**, **PTQ-2F/BTP-3-EH-4Cl**, and **PBDBT-DTBT:PTQ-2F/BTP-3-EH-4Cl** films, where the solid and dashed lines represent the fresh and aged samples’ scattering data in the out-of-plane (Q_*z*_) and in-plane (Q_r_) direction of the films, respectively. The 2D GIWAXS patterns with geometric correction such as the wedge-shape area in 2D have been provided along the out-of-plane direction^69,70^ (see Figure S9). The information of wedge-shaped area can be determined by a Gaussian fit to the peak in the pole figure around the polar angle χ = 90°.^69,70^Figure 6a,b shows the 1D out-of-plane GIWAXS profiles (cake-cut with azimuthally averaged over χ = 90–115°) and in-plane GIWAXS profiles (cake-cut with azimuthally averaged over χ = 155–180°) for the various films. These GIWAXS profiles here are only for qualitative and relative interpretation.

Figure 6a,b indicates that the lamellar structure of the polymer donors in the **PBDBT-DTBT/BTP-3-EH-4Cl** and **PTQ-2F/BTP-3-EH-4Cl** films appear to dominantly adopt the “face-on” orientation, and the **PBDBT-DTBT:PTQ-2F/BTP-3-EH-4Cl** film does not show apparent differences in the crystallization of **PBDBT-DTBT** and **BTP-3-EH-4Cl** from that of **PBDBT-DTBT/BTP-3-EH-4Cl**. Both the (100) diffraction peak intensities of the fresh **PBDBT-DTBT** and **PTQ-2F** films are several times higher than that of the pristine **BTP-3-EH-4Cl** film, representing the dominance of the polymer donors’ crystallization in the blend films (see Figure S10a–d). All of the **PBDBT-DTBT:PTQ-2F/BTP-3-EH-4Cl**, **PBDBT-DTBT/BTP-3-EH-4Cl**, and **PTQ-2F/BTP-3-EH-4Cl** films show that both the intensities of (100) and (010) diffraction peaks decreased after the films underwent thermal aging (under 85 °C for 503 h), suggesting a loss of crystallinity and π–π stacking order and being possibly a contributing factor to the device performance degradation after thermal aging. Based on the GIWAXS profiles and Scherrer’s equation, the crystal correlation length for mainly the donor phase is around 2 nm.

The 1D GISAXS profiles were obtained by a horizontal line-cut around the Yoneda peak (the greatest intensity along the in-plane direction) of the active layer on the 2D GISAXS patterns (Figure S11).^70,71^Figure 6c shows the 1D GISAXS profiles of the fresh and aged (under 85 °C for 503 h) single p–i–n **PBDBT-DTBT/BTP-3-EH-4Cl**, **PTQ-2F/BTP-3-EH-4Cl**, and twin p–i–n **PBDBT-DTBT:PTQ-2F/BTP-3-EH-4Cl** films reduced along the in-plane direction of the 2D diffraction patterns; the low-*q* region (<0.015 Å^–1^) of the pristine **BTP-3-EH-4Cl** film reveals a power-law scattering behavior where the scattering intensity is proportional to *q* following *I*(*q*) ∝ *q*^–*n*^, with *n* equal to 2, indicating that characteristic nanoscale disk-like domains formed by the aggregation or ordered-packing of the acceptor **BTP-3-EH-4Cl** molecules. The fitted radius and thickness of the **BTP-3-EH-4Cl** disk-like domain are 97 and 8.1 nm, respectively. The GISAXS profiles of other pristine and blend films display characteristics of fractal structure factors (power-law intensity behavior in a specific q range), indicating the formation of a fractal-aggregation network, and this network, therefore, cannot be properly fitted with the cylindrical form factor as described in the previous literature.^33,72^

The fractal network model for modeling the morphology of the active layers in OPV was carried out in the literature,^65,73^ and it was recently adopted by several groups for the various morphology of the active layers of OPV materials.^7,62−64,74,75^ The equations and explanations on modeling of the fractal network can be found in the Supporting Information. The low-*q* region (<0.015 Å^–1^) of the fresh **PBDBT-DTBT** and **PTQ-2F** films reveals a power-law scattering with *n* values being between 1.5 and 2.3, showing the characteristic of fractal aggregation from the primary clusters to the large network. Based on the fact that the 1D GISAXS intensities of the fresh **PBDBT-DTBT** and **PTQ-2F** films are at least ten times higher than that of the pristine **BTP-3-EH-4Cl** film, it can be assumed that the scattering intensity in the p–i–n films is mainly contributed by the aggregated polymer domains of **PBDBT-DTBT** and **PTQ-2F**. The intensities of **PBDBT-DTBT/BTP-3-EH-4Cl** and **PTQ-2F/BTP-3-EH-4Cl** p–i–n films are slightly lower than those of the respective fresh donor films, indicating that the crystallization of the **PBDBT-DTBT** or **PTQ-2F** donor is suppressed by incorporating the **BTP-3-EH-4Cl** acceptor phase in the p–i–n films.

The measured GISAXS data points of these pristine and p–i–n structured films can be fitted well by the fractal-like network model using eq S3.^7,62−64,73−75^Table 2 shows the model fitted primary cluster radius (*R*_pc_), correlation length (ξ), domain radius (*R*_g_), and fractal dimension of the network (*D*) values. The *R*_pc_, ξ, *R*_g_, and *D* values for the as-prepared fresh **PBDBT-DTBT** and **PTQ-2F** film are 4.6 30.1, 45.6, and 1.7, respectively, and 3.8 49.9, 96.9, and 2.29, respectively. These two polymer donors have large differences in the correlation length and domain radius values. The *R*_pc_, ξ, *R*_g_, and *D* values for the as-prepared **PBDBT-DTBT/BTP-3-EH-4Cl** single p–i–n film are 3.6 nm, 14.7 nm, 27.6 nm, and 2.2, respectively. The *R*_pc_ and *R*_g_ values (3.6 and 27.6 nm) of the **PBDBT-DTBT** donor phase in the **PBDBT-DTBT/BTP-3-EH-4Cl** p–i–n film are smaller than *R*_pc_ and *R*_g_ values (4.6 and 45.6 nm) in the fresh **PBDBT-DTBT** film, suggesting that the **PBDBT-DTBT** crystallization and network formation were suppressed by the presence of the **BTP-3-EH-4Cl** acceptor phase in the interfacial regions. The *R*_pc_, ξ, *R*_g_, and *D* values for the **PTQ-2F/BTP-3-EH-4Cl** p–i–n film are 4.0 nm, 48.5 nm, 90.3 nm, and 2.18 nm, respectively, almost the same in the case of the fresh **PTQ-2F** film, indicating that the *R*_pc_ and *R*_g_ of the **PTQ-2F** phase are less affected by the **BTP-3-EH-4Cl** acceptor phase in the p-i-n film. The *D* value for the polymer donors ranges from 1.7 to 2.3, suggesting that the fractal network adopted a two-dimensional-like (or disk-like) and open-branch structure.

The *R*_pc_, ξ, *R*_g_, and *D* values for the as-prepared twin p-i-n **PBDBT-DTBT:PTQ-2F/BTP-3-EH-4Cl** film are 3.07, 17.6, 34.7, and 2.33, respectively. Hence, the *R*_pc_ value (3.07 nm) of as-prepared twin p–i–n **PBDBT-DTBT:PTQ-2F/BTP-3-EH-4Cl** film is smaller than the *R*_pc_ value of 3.6 and 4.0 nm for the single p-i-n **PBDBT-DTBT/BTP-3-EH-4Cl** and **PTQ-2F/BTP-3-EH-4Cl** cases, respectively, suggesting that **PBDBT-DTBT** or **PTQ-2F** crystalline clusters have been independent of each other in the twin p–i–n **PBDBT-DTBT:PTQ-2F/BTP-3-EH-4Cl** film during their nucleation and growth. The *R*_g_ value of the as-prepared twin p–i–n **PBDBT-DTBT:PTQ-2F/BTP-3-EH-4Cl** film is 34.7 nm, lying between 27.6 nm for **PBDBT-DTBT/BTP-3-EH-4Cl** and 90.3 nm for **PTQ-2F/BTP-3-EH-4Cl**, close to the average value by weight percentage of **PBDBT-DTBT** and **PTQ-2F** in the film, revealing the formation of network domains in the twin p–i–n **PBDBT-DTBT:PTQ-2F/BTP-3-EH-4Cl** film without mutual interaction between the **PBDBT-DTBT** and **PTQ-2F** crystalline clusters. This result indicates that there are two independent p–i–n structures. The sequence order of *R*_pc_ determined by GISAXS, from the largest to the smallest value, is **PTQ-2F/BTP-3-EH-4Cl** > **PBDBT-DTBT/BTP-3-EH-4Cl** > **PBDBT-DTBT:PTQ-2F/BTP-3-EH-4Cl**, which is consistent with the roughness order obtained by AFM images (Figure S12a–c). The smallest *R*_pc_ of 3.07 nm for the **PBDBT-DTBT:PTQ-2F/BTP-3-EH-4Cl** film can be partially attributed to the larger donor/acceptor interfaces because of the twin p–i–n structure that provides two i-regions such as molecularly mixed **PBDBT-DTBT:BTP-3-EH-4Cl** and **PTQ-2F:BTP-3-EH-4Cl** regions that are favorable for charge separation. It is, therefore, evident that the nanoscale structured primary cluster size (represented by *R*_pc_) within the twin i-region (molecularly mixed region) plays a pivotal role in determining the active layer’s morphology, and the important results of smaller cluster size that surpasses both of the crystallization degree of donor or acceptor phases and the domain size (represented by *R*_g_) of the fractal network indicate the possibility of the presence of the twin p–i–n structure.

To decipher the effect of thermal aging on the morphology of the samples, we also performed GISAXS analyses on the samples and found that the *R*_pc_, ξ, *R*_g_, and *D* values for the aged **PBDBT-DTBT/BTP-3-EH-4Cl** single p–i–n film are 3.4 nm, 20.2 nm, 36.2 nm, and 2.08 nm, respectively, whereas the *R*_pc_, ξ, *R*_g_, and *D* values for the as-prepared **PBDBT-DTBT/BTP-3-EH-4Cl** single p–i–n film are 3.6 nm, 14.7 nm, 27.6 nm, and 2.2, respectively. As compared to the corresponding as-prepared **PBDBT-DTBT/BTP-3-EH-4Cl** film, the thermal annealing process caused *R*_g_ to be enlarged from 27.6 to 36.2 nm. The *R*_pc_, ξ, *R*_g_, and *D* values for the aged **PBDBT-DTBT:PTQ-2F/BTP-3-EH-4Cl** twin p–i–n film are 3.5 nm, 46 nm, 80 nm, and 2.01, respectively. Similarly, the thermal aging process apparently causes the *R*_g_ value to be enlarged from 34.7 (as-prepared **PBDBT-DTBT:PTQ-2F/BTP-3-EH-4Cl** twin p–i–n film) to 80 nm (aged **PBDBT-DTBT:PTQ-2F/BTP-3-EH-4Cl** twin p–i–n film). The results show that the *R*_g_ value increases substantially during the aging process for the twin p–i–n **PBDBT-DTBT:PTQ-2F/BTP-3-EH-4Cl** film more than for the **PBDBT-DTBT/BTP-3-EH-4Cl** film, which indicates a loss of interface between the two donors and also the interface between the donor and acceptor, such that it helps to provide more stability by possibly collapsing the twin p–i–n structure into a single p–i–n structure, a factor that contributes to slowing down the device performance degradation during aging.

Figure 6d depicts the device’s thermal stability by plotting the PCE values of the devices versus thermal aging (*T* = 85 °C) time. The *T*_80_ (time required to reach 80% of the fresh device’s PCE value at 85 °C) lifetime of the **PBDBT-DTBT:PTQ-2F/BTP-3-EH-4Cl** device is 906 h, far superior to the 196 h (**PBDBT-DTBT/BTP-3-EH-4Cl** device) and 284 h (**PTQ-2F/BTP-3-EH-4Cl** devices) of the single p–i–n devices. The critical role in the relationship between the structure and device degradation mechanism is shifted to *R*_g_ instead of *R*_pc_ for the following reasons. The reason for the higher *T*_80_ lifetime of the aged **PBDBT-DTBT:PTQ-2F/BTP-3-EH-4Cl** device can be attributed to its large *R*_g_ because of the twin p–i–n structure, favorable for charge transport in the interpenetrated network, and the critical factor in the structure–performance relationship for the degradation mechanism shifts from the cluster size to the domain size, as demonstrated by the results presented in Figure 6c and Table 3.

**Table 3:** GISAXS Fitting Parameters are Comprehensively Detailed, Encompassing the Mean Radius (*R*_pc_), Correlation Length (ξ), Domain Radius (*R*_g_), and Fractal Dimension of the Network (*D*)

active layer	*R*_pc_ (nm)	ξ (nm)	*R*_g_ (nm)	*D*
**PBDBT-DTBT** (as-prepared)	4.6	30.1	45.6	1.7
**PTQ-2F** (as-prepared)	3.8	49.9	96.9	2.29
**PBDBT-DTBT/BTP-3-EH-4Cl** (as-prepared)	3.6	14.7	27.6	2.2
**PTQ-2F/BTP-3-EH-4Cl** (as-prepared)	4.0	48.5	90.3	2.18
**PBDBT-DTBT:PTQ-2F/BTP-3-EH-4Cl** (as-prepared)	3.07	17.6	34.7	2.33
**PBDBT-DTBT/BTP-3-EH-4Cl** (aged)	3.4	20.2	36.2	2.08
**PBDBT-DTBT:PTQ-2F/BTP-3-EH-4Cl** (aged)	3.5	46	80	2.01

## 4 Conclusions

In summary, we have developed OPVs with a twin p–i–n **PBDBT-DTBT:PTQ-2F/BTP-3-EH-4Cl** active layer having a champion PCE of 18.6%, significantly improved over the 16.4% for the control BHJ ternary blend **PBDBT-DTBT:PTQ-2F:BTP-3-EH-4Cl** device and the PCEs of 16.6 and 13.5% for the single p–i–n structured **PBDBT-DTBT/BTP-3-EH-4Cl** and **PTQ-2F/BTP-3-EH-4Cl** devices, respectively. Moreover, the PCE values of the single p–i–n structured **PBDBT-DTBT/BTP-3-EH-4Cl** and **PTQ-2F/BTP-3-EH-4Cl** devices are in turn higher than the PCE values of 16.1 and 7.6% for the BHJ binary blend **PBDBT-DTBT:BTP-3-EH-4Cl** and binary blend **PTQ-2F:BTP-3-EH-4Cl** devices, respectively. We confirmed the p–i–n structured active layers with TOF–SIMS depth profiling and GISAXS analyses, and the results suggest that the **BTP-3-EH-4Cl** acceptors have diffused within the **PBDBT-DTBT:PTQ-2F** polymer donors to form substantial interfacial regions, but still maintaining its pristine state in a small region, providing p–n junction for efficient excitons dissociation, and establishing vertically continuous pathways for charge transport through the twin p–i–n structured active layer. Under thermal aging at 85 °C, the *T*_80_ lifetime of the twin p–i–n **PBDBT-DTBT:PTQ-2F/BTP-3-EH-4Cl** device is 906 h, far surpassing the 196 h for the single p–i–n **PBDBT-DTBT/BTP-3-EH-4Cl** device and 284 h for the single p–i–n **PTQ-2F/BTP-3-EH-4Cl** device. The improvement is attributed to the large domain size in the twin p–i–n structured **PBDBT-DTBT:PTQ-2F/BTP-3-EH-4Cl** device, which created a favorable interpenetrated network in the i-region for charge transport.

## Supporting Information Available

Sequential deposition process, UV, the dark CELIV j–t profiles, SCLC, the light intensity dependent on *V*_oc_, FTPS-EQE, and EL, contour plots of TA, 2D GIWAXS, and atomic force microscopy (PDF)
