# Supplementary material for: Achieving High Efficiency and Stability in Organic Photovoltaics with a Nanometer-Scale Twin p–i–n Structured Active Layer
Source: ACS Appl Mater Interfaces. 2024 Jul 23;16(31):41244–56. doi: 10.1021/acsami.4c08868 (PMC11311131; doi:10.1021/acsami.4c08868)
Supplement: Supplementary file 1 — am4c08868_si_001.pdf [file am4c08868_si_001.pdf]

Supporting Information

Achieving High Efficiency and Stability in Organic Photovoltaics with a Nanometer-Scale Twin p-i-n Structured Active Layer

Bin Chang,[a] *Bing-Huang Jiang,*[b] *Chih-Ping Chen,*[b,c] *Kai Chen,*[d,e,f] *Bo-Han Chen,*[g] *Shaun Tan,*[h] *Tzu-Ching Lu,*[a] *Cheng-Si Tsao,*[i,j] *Yu-Wei Su,*[k] *Shang-Da Yang,*[g] *Cheng-Sheng Chen*[a] *and Kung-Hwa Wei**[,a]

[a] *Department of Materials Science and Engineering, National Yang Ming Chiao Tung University, Hsinchu 30010, Taiwan*

[b] *Department of Materials Engineering, Ming Chi University of Technology, New Taipei City 243303, Taiwan*

[c] *College of Engineering, Chang Gung University, Taoyuan 33302, Taiwan*

[d] *Robinson Research Institute, Victoria University of Wellington, New Zealand*

[e] *MacDiarmid Institute for Advanced Materials and Nanotechnology, New Zealand*

[f] *The Dodd-Walls Centre for Photonic and Quantum Technologies, New Zealand*

[g] *Institute of Photonics Technologies, National Tsing Hua University, Hsinchu 300044, Taiwan*

[h] *Department of Chemistry, Massachusetts Institute of Technology, Cambridge, MA 02139, USA*

[i] *Department of Materials Science and Engineering, National Taiwan University, Taipei 10617, Taiwan*

[j] *National Synchrotron Radiation Research Center, Hsinchu 30010, Taiwan*

[k] *Department of Molecular Science and Engineering, Institute of Organic and Polymeric Materials, National Taipei University of Technology, Taipei 10608, Taiwan.*

Corresponding Author

*E-mail: khwei@nycu.edu.tw

Supplementary Equation

Carrier Mobility (μ) mesured with the Photo-CELIV Method

$$\mu = \frac{2d^2}{3At_{max}^2\left[1+0.36\frac{\Delta j}{j(0)}\right]} \quad (1)$$

which, d represents the thickness of the active layer, t_{max} is the time taken to reach the maximum extraction current, A signifies the rise rate of the applied voltage pulse, and Δj and $j(0)$ correspond to the shift and the initial current step, respectively.

Model Fitting for GISAXS curves

The GISAXS intensity caused by the fractal network consisting of primary clusters of **PBDBT-DTBT** or **PTQ-2F** crystallites can be expressed as

$$I(q) = P(q) \cdot S(q) \quad (2)$$

$$S(q) = 1 + \frac{sin\left[(D-1)tan^{-1}(q\xi)\right]}{(qR_{pc})^D} \cdot \frac{D\Gamma(D-1)}{\left[1+\frac{1}{(q\xi)^2}\right]^{\frac{D-1}{2}}} \quad (3)$$

where $P(q)$ in equation (2) is the form factor of spherical primary clusters formed by donor molecules with a mean radius R_{pc}. In equation (3), $S(q)$ is the structure factor of fractal-like network formed by aggregation of primary clusters having a Schulz size distribution. The ξ is the correlation length (or characteristic length) of fractal-like network. D is the fractal dimension of network and Γ is a gamma function. The domain size of this fractal network can be approximately characterized by the radius of gyration $R_g = \xi[D(D+1)/2]^{1/2}$. The least-squares fitting of equation (3) to GISAXS profile determines the values of R_{PC}, ξ and D.

Supplementary Figures

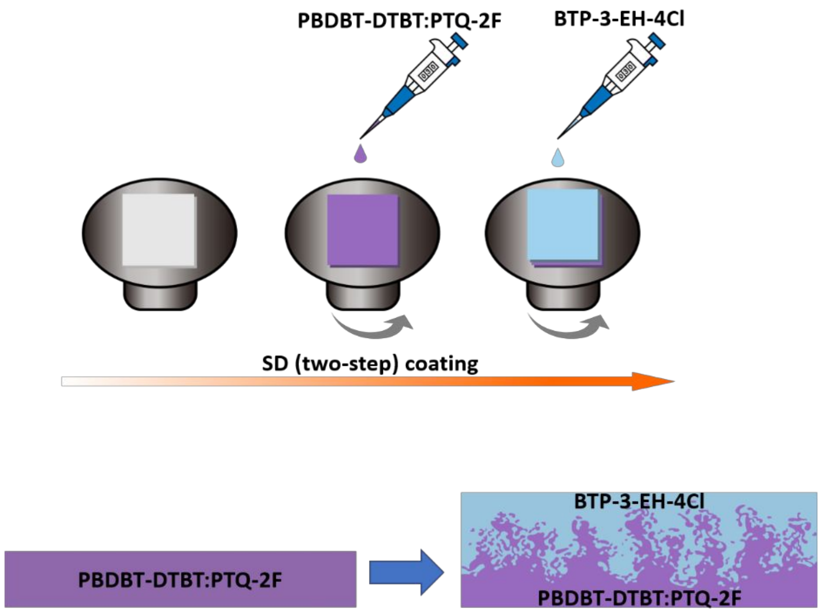

Figure S1. Schematic representation of the sequential deposition process with a spin-coating method and the formation of twin p-i-n structures.

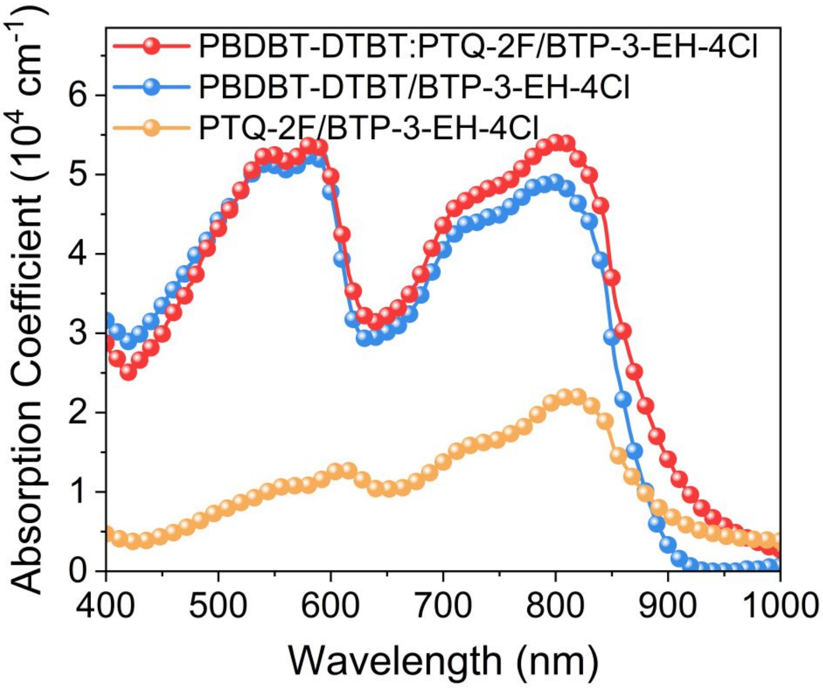

Figure S2. The optical absorption coefficient of all films at the active layers' compositions.

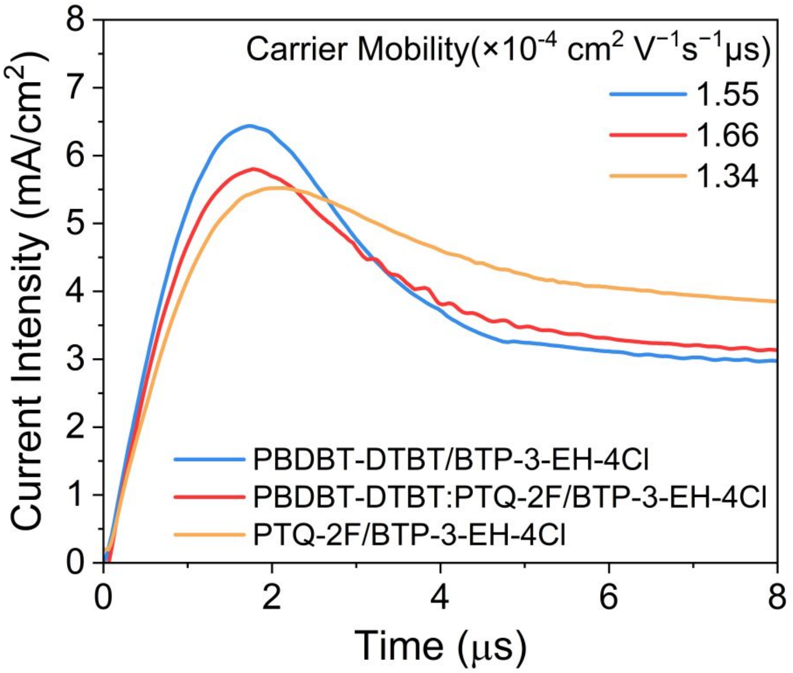

Figure S3. The dark CELIV j–t profiles (carrier mobility) of OPVs devices with different p-i-n structures.

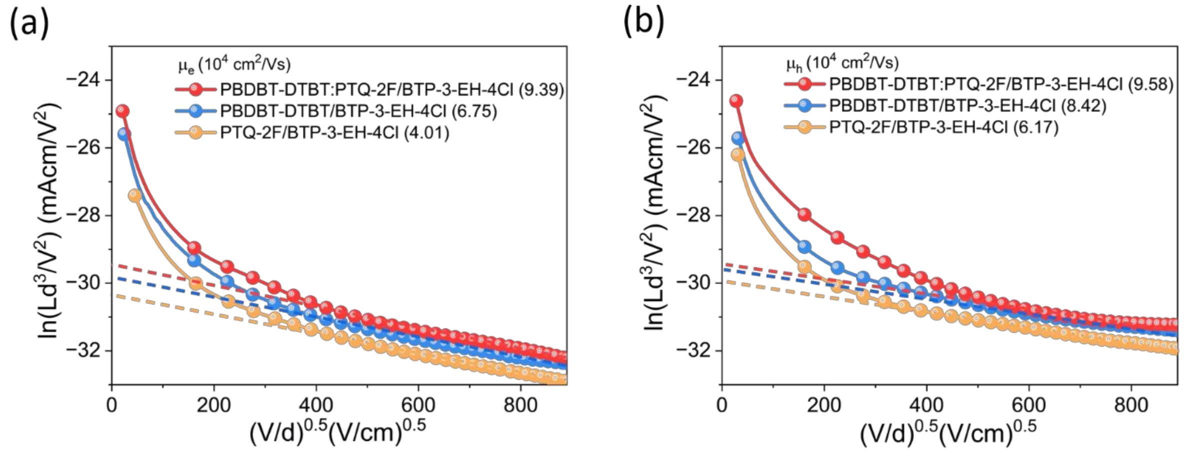

Figure S4. (a) Electron mobility (μ_e) and (b) hole mobility (μ_h) of OPVs devices with different p-i-n structures using SCLC measurements.

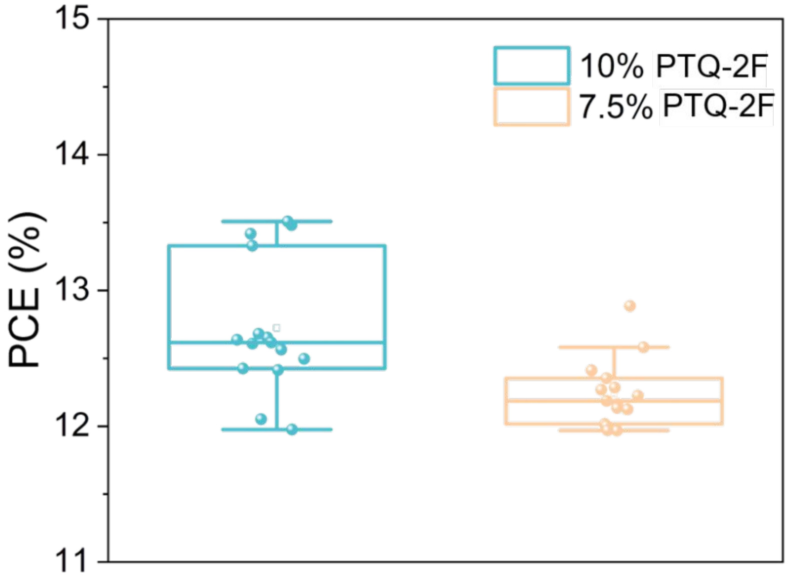

Figure S5. Box plots showing the distribution of the power conversion efficiencies (PCEs) of the **PBDBT-DTBT:PTQ-2F/BTP-3-EH-4Cl** devices at different ratios of **PBDBT-DTBT:PTQ-2F**

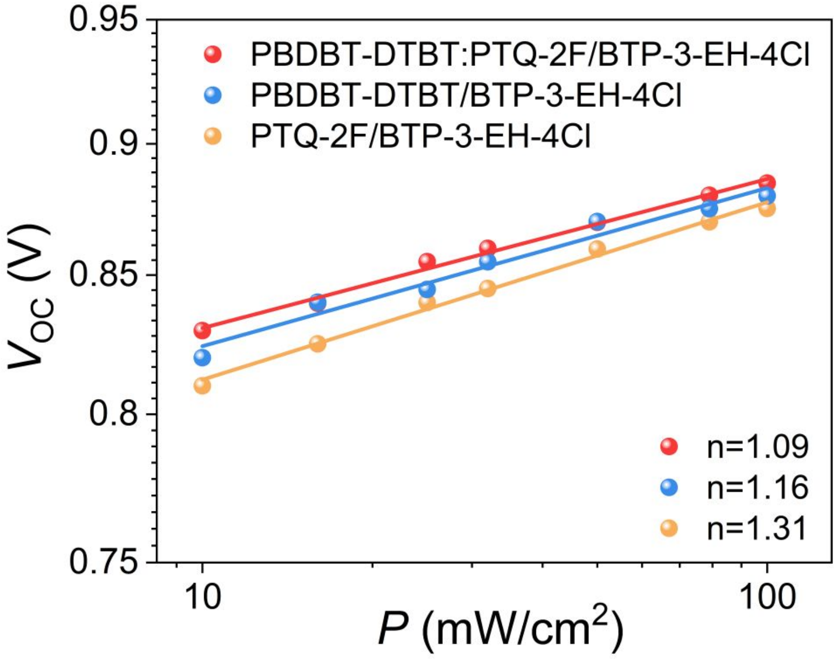

Figure S6. The dependence of *V*oc of the devices on the incident light intensity.

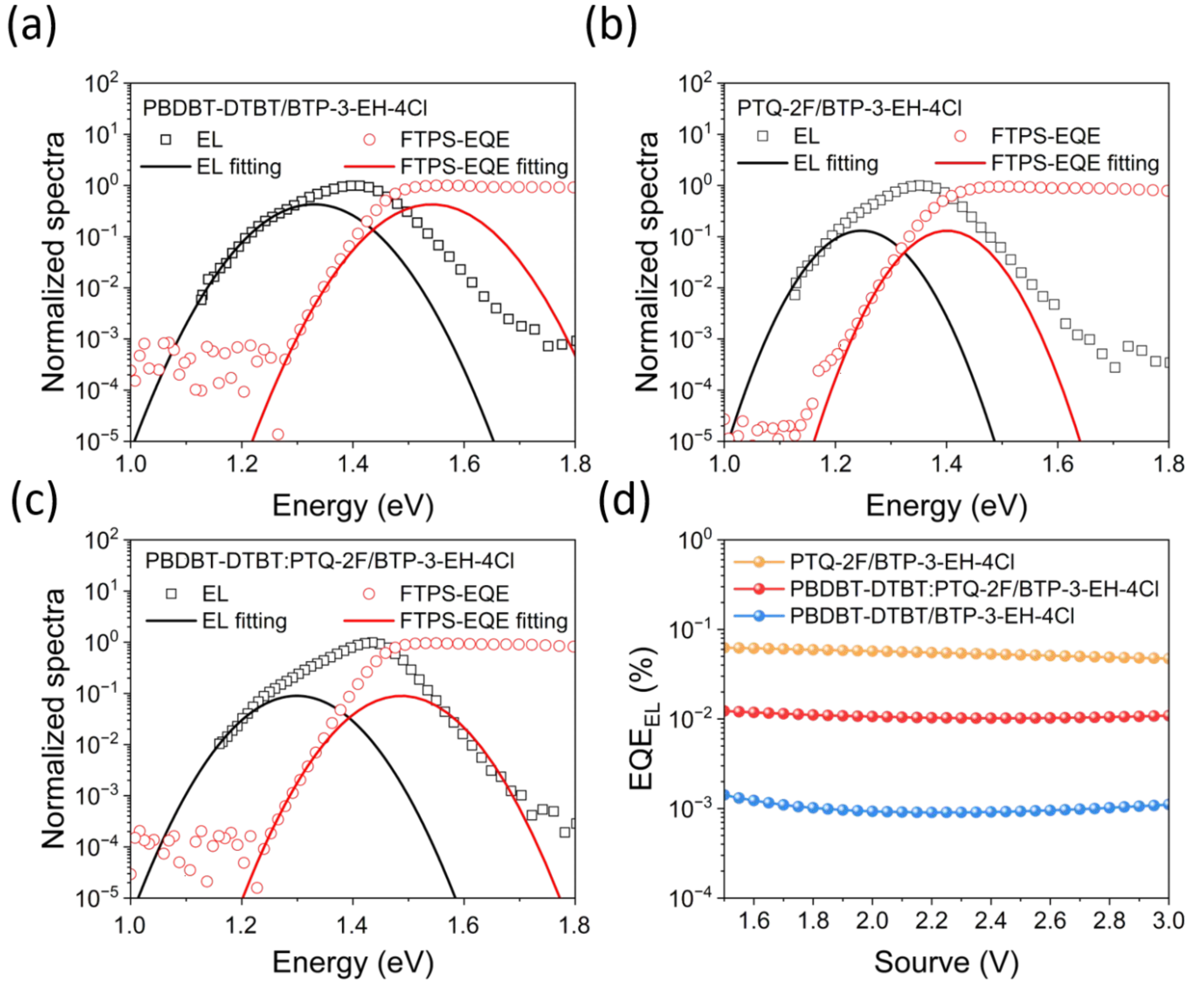

Figure S7. The FTPS-EQE and EL of (a) **PBDBT-DTBT/BTP-3-EH-4Cl**, (b) **PTQ-2F/BTP-3-EH-4Cl**, and (c) **PBDBT-DTBT:PTQ-2F/BTP-3-EH-4Cl** and (d) EQE_{EL} of the p-i-n OPV devices.

Table S1. Summary of energy loss parameters of SD devices measured and calculated from FTPS-EQE and EL.

	E_g [eV]	V_{OC} [V]	V_{loss} [V]	E_{CT}	ΔE_{CT} [eV]	ΔE_{rad} [V]	$\Delta E_{non-rad}$ [V]	EQE_{EL}
PBDBT-DTBT/BTP-3-EH-4Cl	1.465	0.86	0.605	1.436	0.029	0.279	0.297	9.63×10^{-6}
PTQ-2F/BTP-3-EH-	1.407	0.85	0.657	1.324	0.083	0.28	0.194	5.20×10^{-6}

4Cl								
PBDBT-DTBT:PTQ-2F/BTP-3-EH-4Cl	1.468	0.88	0.588	1.393	0.075	0.278	0.235	1.04×10^{-4}

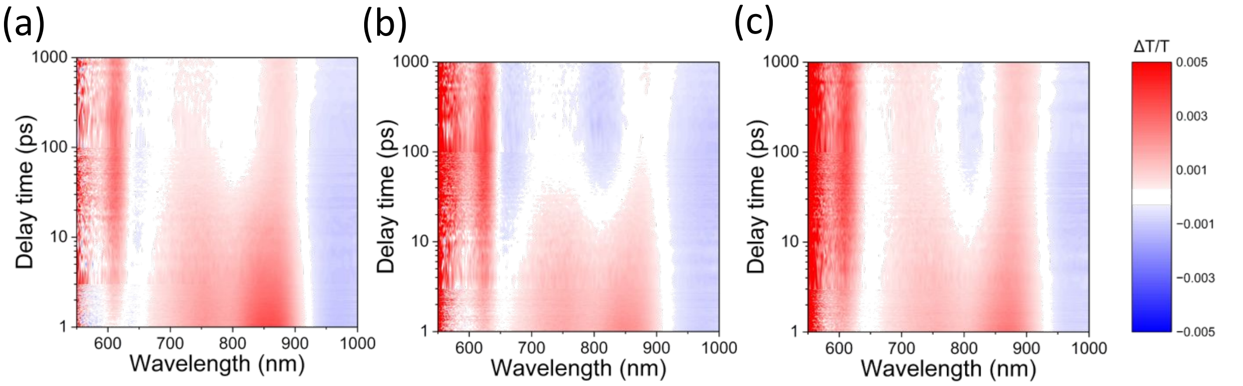

Figure S8. Contour plots of the time-resolved TA spectra for (a) **PBDBT-DTBT/BTP-3-EH-4Cl**, (b) **PTQ-2F/BTP-3-EH-4Cl**, and (c) **PBDBT-DTBT:PTQ-2F/BTP-3-EH-4Cl** films.

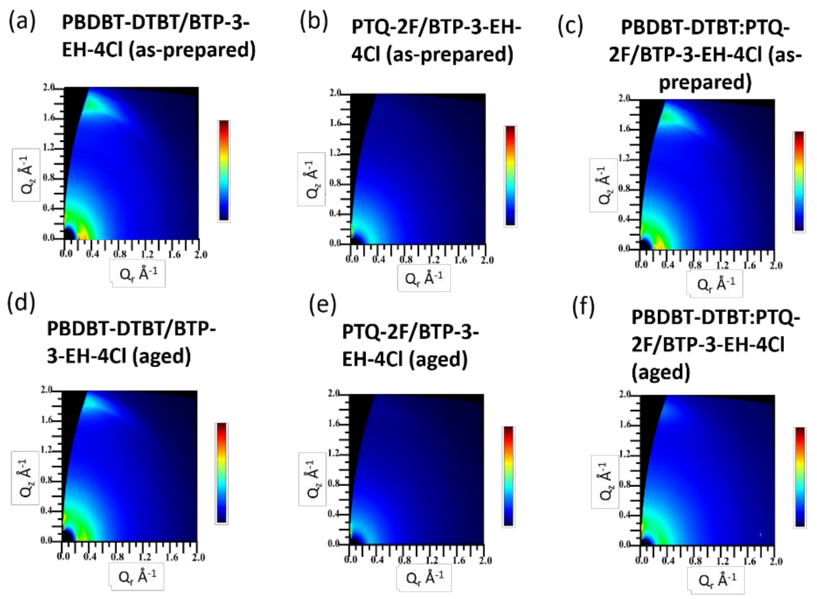

Figure S9. 2D GIWAXS of the pristine p-i-n films of (a) **PBDBT-DTBT/BTP-3-EH-4Cl**, (b)

PTQ-2F/BTP-3-EH-4Cl, and (c) **PBDBT-DTBT:PTQ-2F/BTP-3-EH-4Cl**. 2D GIWAXS of the aged p-i-n films of (d) **PBDBT-DTBT/BTP-3-EH-4Cl**, (e) **PTQ-2F/BTP-3-EH-4Cl**, and (f) **PBDBT-DTBT:PTQ-2F/BTP-3-EH-4Cl**.

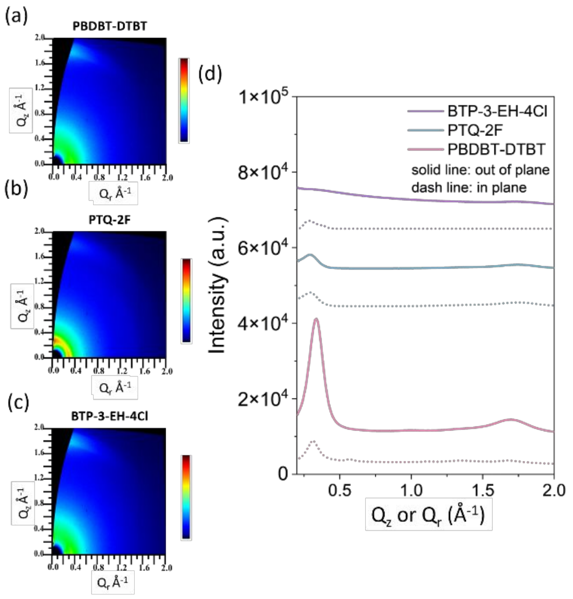

Figure S10. 2D GIWAXS patterns of the pristine (a) **PBDBT-DTBT**, (b) **PTQ-2F**, and (c) **BTP-3-EH-4Cl** films. (d) Corresponding 1D out-of-plane GIWAXS profiles of **PBDBT-DTBT**, **PTQ-2F**, and **BTP-3-EH-4Cl** films.

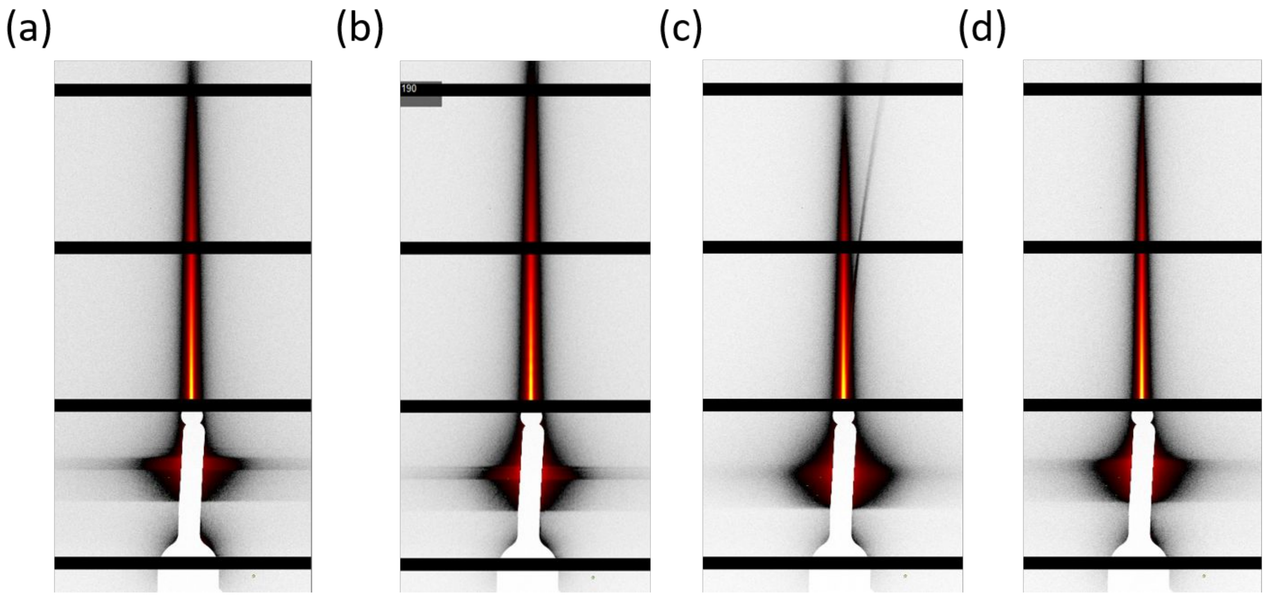

Figure S11. 2D GISAXS patterns of (a) **PBDBT-DTBT/BTP-3-EH-4Cl** (as prepare), (b) **PBDBT-**

DTBT:PTQ-2F/BTP-3-EH-4Cl (as prepare), (c) **PBDBT-DTBT/BTP-3-EH-4Cl** (aged), and (d) **PBDBT-DTBT:PTQ-2F/BTP-3-EH-4Cl** (aged).

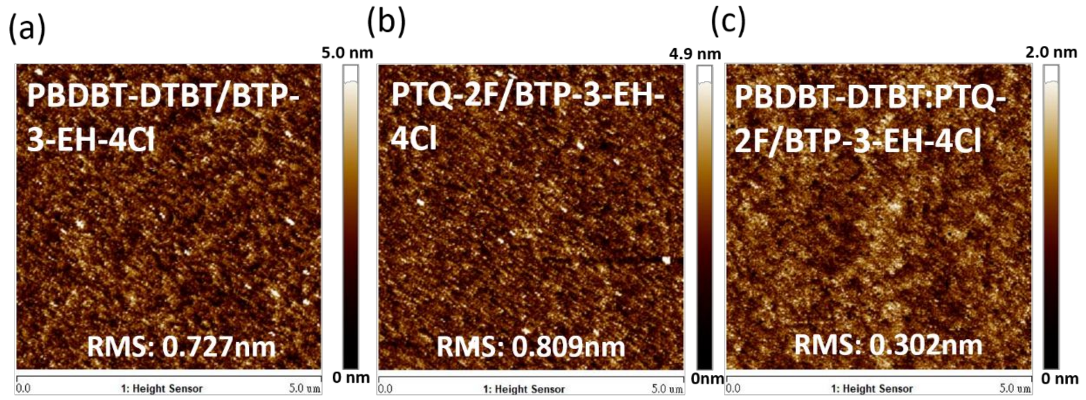

Figure S12. Atomic Force Microscopy topology images of (a) **PBDBT-DTBT/BTP-3-EH-4Cl**, (b) **PTQ-2F/BTP-3-EH-4Cl**, and (c) **PBDBT-DTBT:PTQ-2F/BTP-3-EH-4Cl** films.
